# NETWORK ANALYSIS OF HEALTH PROBLEMS AMONG OLDER WORKERS AGED 65–75 IN KOREA

**DOI:** 10.1093/geroni/igad104.2426

**Published:** 2023-12-21

**Authors:** Layoung Kim, Gwang Suk Kim, Min Kyung Park, Jae Jun Lee

**Affiliations:** Graduate School of Yonsei University, Seoul, Republic of Korea; Yonsei University College of Nursing, Seoul, Republic of Korea; Graduate School of Yonsei University, Seoul, Republic of Korea; Yonsei University College of Nursing, Seoul, Republic of Korea

## Abstract

Korea’s effective age of labor market exit is 72.3 years the longest age among the Organisation for Economic Co-operation and Development (OECD). The increase in the number and age of older workers can result in the burden of workplace safety and health management. Health policies and programs can be efficient only when they are established based on a specific understanding of health problems by occupational groups. In this study, we identified older workers’ central health problems. Six health problems were reported by the 6th Korean Working Conditions Survey. The occupational characteristics were distributed into three groups: white-, blue-, and pink-collar. The data of older workers aged 65-75 who worked over 40 hours per week (N=1,086) was used for network analysis through the bootnet and qgraph packages in the R software. The health problems reported by older workers were: backache (39.9%); muscular pains in the shoulder, neck, and upper limb (39.7%); muscular pains in the lower limb (24.1%); fatigue (31.2%); headaches and eyestrain (17.3%); and anxiety (6.5%). The most central node of network was muscular pains in upper, which had significant relationships with backache, pains in lower, and fatigue. The correlation stability coefficient of strength was 0.672, which was preferable. In the subdivide, the pink-collar group showed muscular pains in the lower limb as the highest centrality. Since older people have simultaneous health problems, healthcare management should consider the relationships between problems. Additionally, centrality differed from occupational groups. These findings can be applicated to provide customized programs for the older workers.

